# Supplementary material for: The evaluation of indoxyl sulfate in the general population in Kanegasaki Iwate: A cross-sectional study (KANEGASAKI study)
Source: PLoS One. 2025 Dec 17;20(12):e0332655. doi: 10.1371/journal.pone.0332655 (PMC12711065; doi:10.1371/journal.pone.0332655)
Supplement: S1 Table — (PPTX) [file pone.0332655.s001.pptx]

## Slide 1
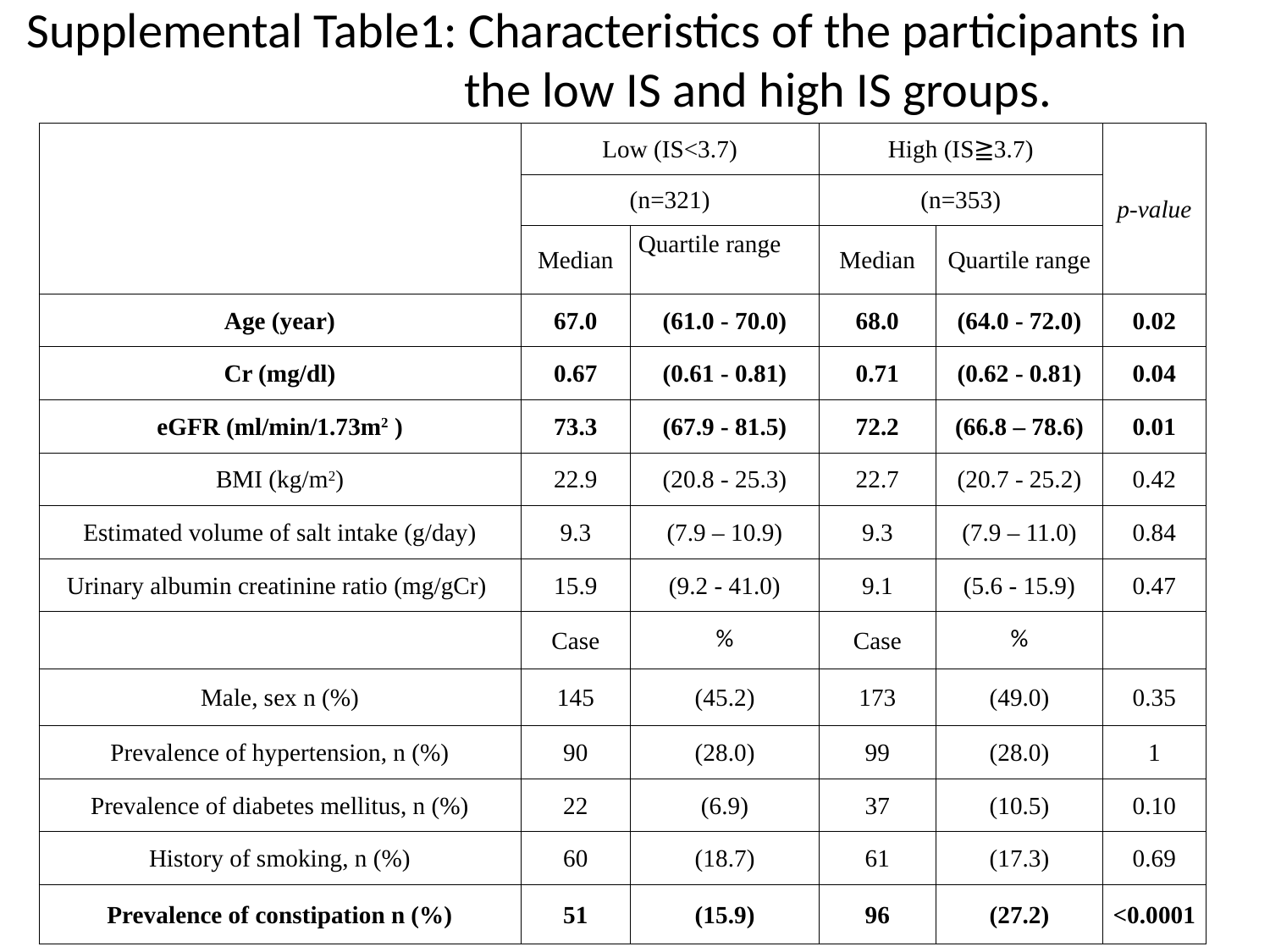

Supplemental Table1: Characteristics of the participants in
 the low IS and high IS groups.
| | Low (IS<3.7) | | High (IS≧3.7) | | p-value |
| --- | --- | --- | --- | --- | --- |
| | (n=321) | | (n=353) | | |
| | Median | Quartile range | Median | Quartile range | |
| Age (year) | 67.0 | (61.0 - 70.0) | 68.0 | (64.0 - 72.0) | 0.02 |
| Cr (mg/dl) | 0.67 | (0.61 - 0.81) | 0.71 | (0.62 - 0.81) | 0.04 |
| eGFR (ml/min/1.73m2 ) | 73.3 | (67.9 - 81.5) | 72.2 | (66.8 – 78.6) | 0.01 |
| BMI (kg/m2) | 22.9 | (20.8 - 25.3) | 22.7 | (20.7 - 25.2) | 0.42 |
| Estimated volume of salt intake (g/day) | 9.3 | (7.9 – 10.9) | 9.3 | (7.9 – 11.0) | 0.84 |
| Urinary albumin creatinine ratio (mg/gCr) | 15.9 | (9.2 - 41.0) | 9.1 | (5.6 - 15.9) | 0.47 |
| | Case | % | Case | % | |
| Male, sex n (%) | 145 | (45.2) | 173 | (49.0) | 0.35 |
| Prevalence of hypertension, n (%) | 90 | (28.0) | 99 | (28.0) | 1 |
| Prevalence of diabetes mellitus, n (%) | 22 | (6.9) | 37 | (10.5) | 0.10 |
| History of smoking, n (%) | 60 | (18.7) | 61 | (17.3) | 0.69 |
| Prevalence of constipation n (%) | 51 | (15.9) | 96 | (27.2) | <0.0001 |
