# Supplementary material for: The evaluation of indoxyl sulfate in the general population in Kanegasaki Iwate: A cross-sectional study (KANEGASAKI study)
Source: PLoS One. 2025 Dec 17;20(12):e0332655. doi: 10.1371/journal.pone.0332655 (PMC12711065; doi:10.1371/journal.pone.0332655)
Supplement: S2 Table — (PPTX) [file pone.0332655.s002.pptx]

## Slide 1
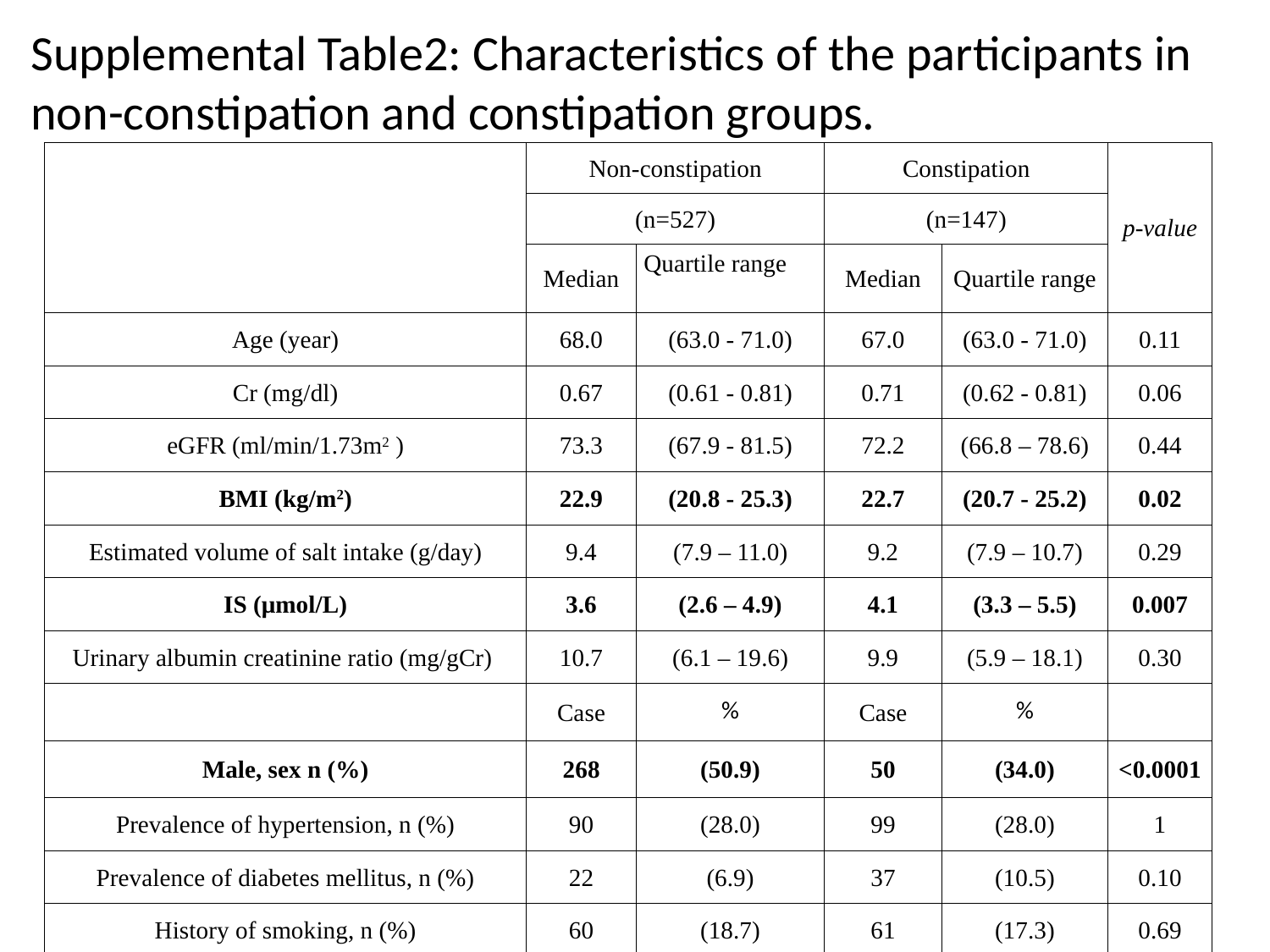

Supplemental Table2: Characteristics of the participants in non-constipation and constipation groups.
| | Non-constipation | | Constipation | | p-value |
| --- | --- | --- | --- | --- | --- |
| | (n=527) | | (n=147) | | |
| | Median | Quartile range | Median | Quartile range | |
| Age (year) | 68.0 | (63.0 - 71.0) | 67.0 | (63.0 - 71.0) | 0.11 |
| Cr (mg/dl) | 0.67 | (0.61 - 0.81) | 0.71 | (0.62 - 0.81) | 0.06 |
| eGFR (ml/min/1.73m2 ) | 73.3 | (67.9 - 81.5) | 72.2 | (66.8 – 78.6) | 0.44 |
| BMI (kg/m2) | 22.9 | (20.8 - 25.3) | 22.7 | (20.7 - 25.2) | 0.02 |
| Estimated volume of salt intake (g/day) | 9.4 | (7.9 – 11.0) | 9.2 | (7.9 – 10.7) | 0.29 |
| IS (μmol/L) | 3.6 | (2.6 – 4.9) | 4.1 | (3.3 – 5.5) | 0.007 |
| Urinary albumin creatinine ratio (mg/gCr) | 10.7 | (6.1 – 19.6) | 9.9 | (5.9 – 18.1) | 0.30 |
| | Case | % | Case | % | |
| Male, sex n (%) | 268 | (50.9) | 50 | (34.0) | <0.0001 |
| Prevalence of hypertension, n (%) | 90 | (28.0) | 99 | (28.0) | 1 |
| Prevalence of diabetes mellitus, n (%) | 22 | (6.9) | 37 | (10.5) | 0.10 |
| History of smoking, n (%) | 60 | (18.7) | 61 | (17.3) | 0.69 |
