# Supplementary material for: The evaluation of indoxyl sulfate in the general population in Kanegasaki Iwate: A cross-sectional study (KANEGASAKI study)
Source: PLoS One. 2025 Dec 17;20(12):e0332655. doi: 10.1371/journal.pone.0332655 (PMC12711065; doi:10.1371/journal.pone.0332655)
Supplement: S3 Table — (PPTX) [file pone.0332655.s003.pptx]

## Slide 1
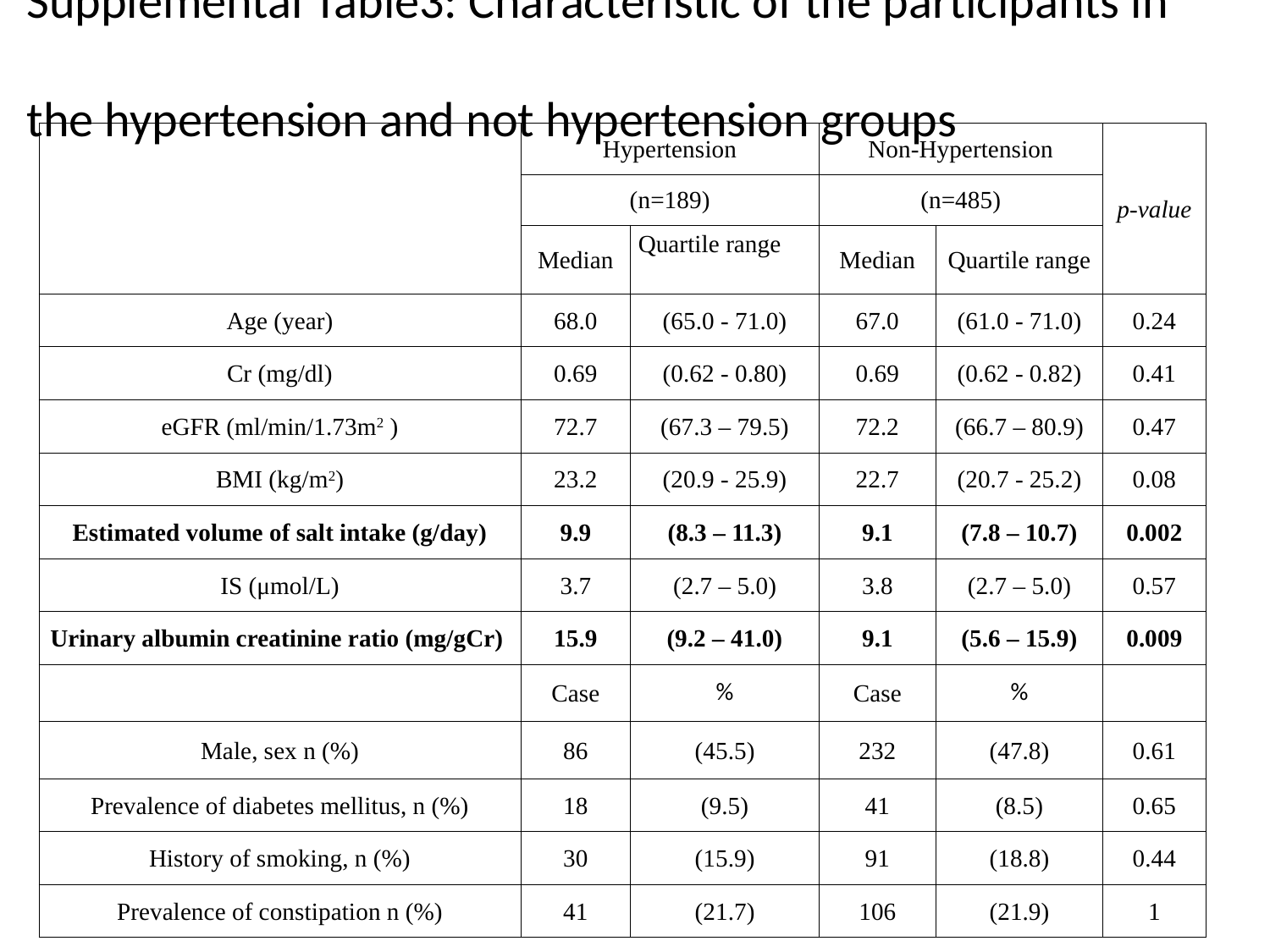

Supplemental Table3: Characteristic of the participants in
the hypertension and not hypertension groups
| | Hypertension | | Non-Hypertension | | p-value |
| --- | --- | --- | --- | --- | --- |
| | (n=189) | | (n=485) | | |
| | Median | Quartile range | Median | Quartile range | |
| Age (year) | 68.0 | (65.0 - 71.0) | 67.0 | (61.0 - 71.0) | 0.24 |
| Cr (mg/dl) | 0.69 | (0.62 - 0.80) | 0.69 | (0.62 - 0.82) | 0.41 |
| eGFR (ml/min/1.73m2 ) | 72.7 | (67.3 – 79.5) | 72.2 | (66.7 – 80.9) | 0.47 |
| BMI (kg/m2) | 23.2 | (20.9 - 25.9) | 22.7 | (20.7 - 25.2) | 0.08 |
| Estimated volume of salt intake (g/day) | 9.9 | (8.3 – 11.3) | 9.1 | (7.8 – 10.7) | 0.002 |
| IS (μmol/L) | 3.7 | (2.7 – 5.0) | 3.8 | (2.7 – 5.0) | 0.57 |
| Urinary albumin creatinine ratio (mg/gCr) | 15.9 | (9.2 – 41.0) | 9.1 | (5.6 – 15.9) | 0.009 |
| | Case | % | Case | % | |
| Male, sex n (%) | 86 | (45.5) | 232 | (47.8) | 0.61 |
| Prevalence of diabetes mellitus, n (%) | 18 | (9.5) | 41 | (8.5) | 0.65 |
| History of smoking, n (%) | 30 | (15.9) | 91 | (18.8) | 0.44 |
| Prevalence of constipation n (%) | 41 | (21.7) | 106 | (21.9) | 1 |
