# Supplementary material for: The evaluation of indoxyl sulfate in the general population in Kanegasaki Iwate: A cross-sectional study (KANEGASAKI study)
Source: PLoS One. 2025 Dec 17;20(12):e0332655. doi: 10.1371/journal.pone.0332655 (PMC12711065; doi:10.1371/journal.pone.0332655)
Supplement: S4 Table — (PPTX) [file pone.0332655.s004.pptx]

## Slide 1
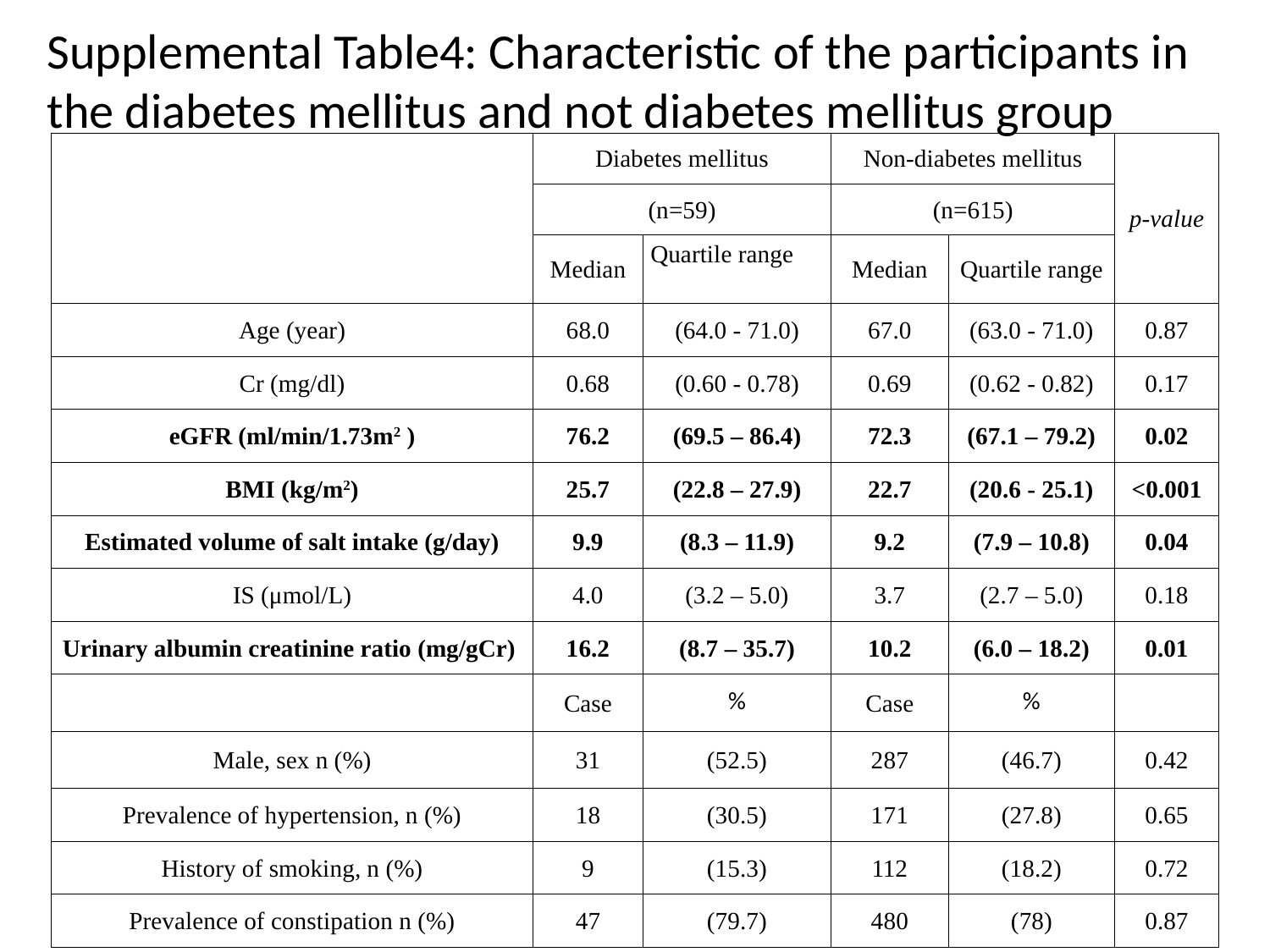

Supplemental Table4: Characteristic of the participants in the diabetes mellitus and not diabetes mellitus group
| | Diabetes mellitus | | Non-diabetes mellitus | | p-value |
| --- | --- | --- | --- | --- | --- |
| | (n=59) | | (n=615) | | |
| | Median | Quartile range | Median | Quartile range | |
| Age (year) | 68.0 | (64.0 - 71.0) | 67.0 | (63.0 - 71.0) | 0.87 |
| Cr (mg/dl) | 0.68 | (0.60 - 0.78) | 0.69 | (0.62 - 0.82) | 0.17 |
| eGFR (ml/min/1.73m2 ) | 76.2 | (69.5 – 86.4) | 72.3 | (67.1 – 79.2) | 0.02 |
| BMI (kg/m2) | 25.7 | (22.8 – 27.9) | 22.7 | (20.6 - 25.1) | <0.001 |
| Estimated volume of salt intake (g/day) | 9.9 | (8.3 – 11.9) | 9.2 | (7.9 – 10.8) | 0.04 |
| IS (μmol/L) | 4.0 | (3.2 – 5.0) | 3.7 | (2.7 – 5.0) | 0.18 |
| Urinary albumin creatinine ratio (mg/gCr) | 16.2 | (8.7 – 35.7) | 10.2 | (6.0 – 18.2) | 0.01 |
| | Case | % | Case | % | |
| Male, sex n (%) | 31 | (52.5) | 287 | (46.7) | 0.42 |
| Prevalence of hypertension, n (%) | 18 | (30.5) | 171 | (27.8) | 0.65 |
| History of smoking, n (%) | 9 | (15.3) | 112 | (18.2) | 0.72 |
| Prevalence of constipation n (%) | 47 | (79.7) | 480 | (78) | 0.87 |
