# Supplementary material for: The evaluation of indoxyl sulfate in the general population in Kanegasaki Iwate: A cross-sectional study (KANEGASAKI study)
Source: PLoS One. 2025 Dec 17;20(12):e0332655. doi: 10.1371/journal.pone.0332655 (PMC12711065; doi:10.1371/journal.pone.0332655)
Supplement: S5 Table — (PPTX) [file pone.0332655.s005.pptx]

## Slide 1
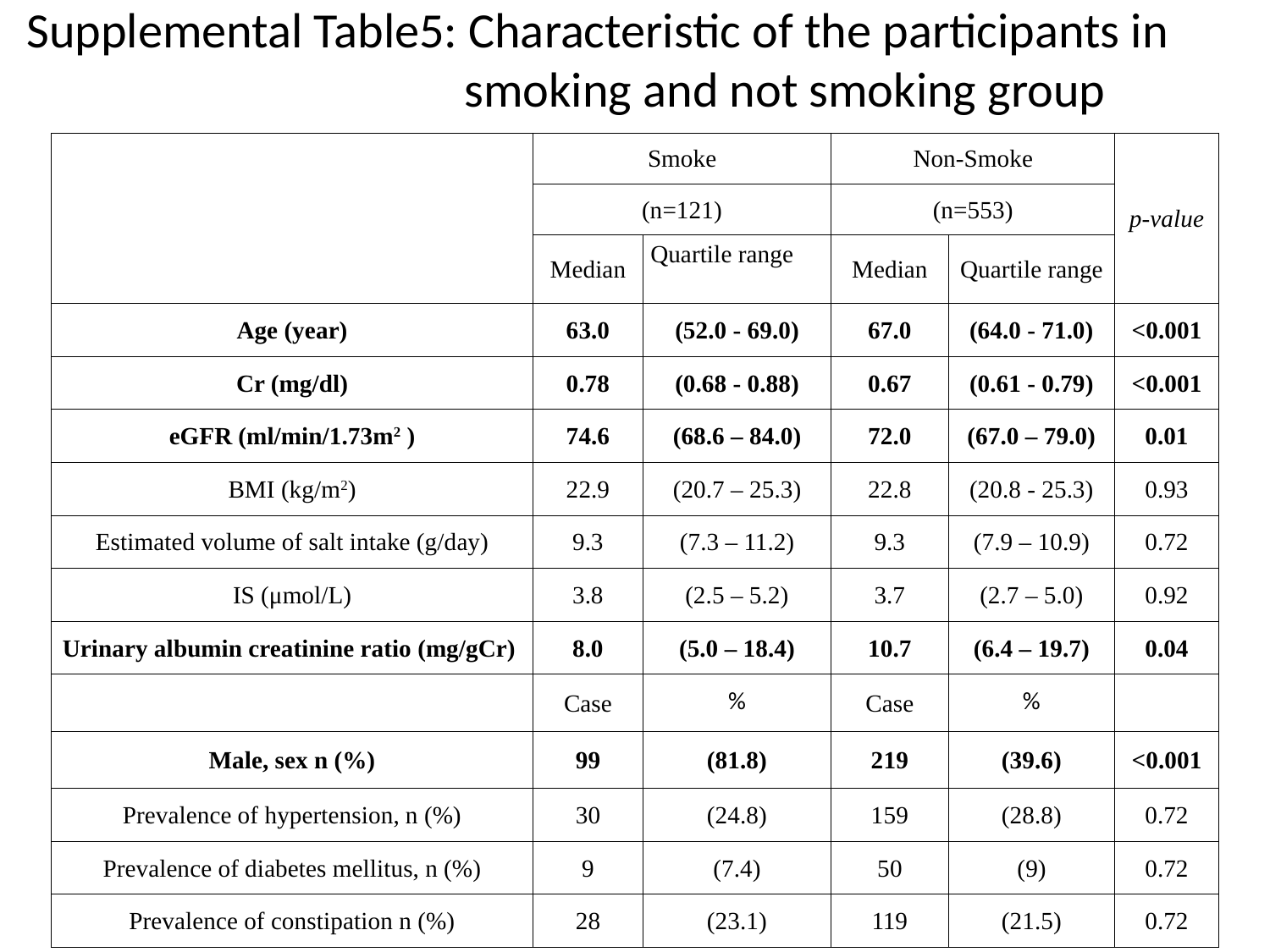

Supplemental Table5: Characteristic of the participants in
 smoking and not smoking group
| | Smoke | | Non-Smoke | | p-value |
| --- | --- | --- | --- | --- | --- |
| | (n=121) | | (n=553) | | |
| | Median | Quartile range | Median | Quartile range | |
| Age (year) | 63.0 | (52.0 - 69.0) | 67.0 | (64.0 - 71.0) | <0.001 |
| Cr (mg/dl) | 0.78 | (0.68 - 0.88) | 0.67 | (0.61 - 0.79) | <0.001 |
| eGFR (ml/min/1.73m2 ) | 74.6 | (68.6 – 84.0) | 72.0 | (67.0 – 79.0) | 0.01 |
| BMI (kg/m2) | 22.9 | (20.7 – 25.3) | 22.8 | (20.8 - 25.3) | 0.93 |
| Estimated volume of salt intake (g/day) | 9.3 | (7.3 – 11.2) | 9.3 | (7.9 – 10.9) | 0.72 |
| IS (μmol/L) | 3.8 | (2.5 – 5.2) | 3.7 | (2.7 – 5.0) | 0.92 |
| Urinary albumin creatinine ratio (mg/gCr) | 8.0 | (5.0 – 18.4) | 10.7 | (6.4 – 19.7) | 0.04 |
| | Case | % | Case | % | |
| Male, sex n (%) | 99 | (81.8) | 219 | (39.6) | <0.001 |
| Prevalence of hypertension, n (%) | 30 | (24.8) | 159 | (28.8) | 0.72 |
| Prevalence of diabetes mellitus, n (%) | 9 | (7.4) | 50 | (9) | 0.72 |
| Prevalence of constipation n (%) | 28 | (23.1) | 119 | (21.5) | 0.72 |
