# Supplementary material for: The evaluation of indoxyl sulfate in the general population in Kanegasaki Iwate: A cross-sectional study (KANEGASAKI study)
Source: PLoS One. 2025 Dec 17;20(12):e0332655. doi: 10.1371/journal.pone.0332655 (PMC12711065; doi:10.1371/journal.pone.0332655)
Supplement: Attachment — Submitted filename: PONE-D-25-26168 Comments.pdf [file pone.0332655.s007.pdf]

PONE-D-25-26168 Comments

Statistical Analysis

1.

One major issue is the data availability. It's claimed by the authors that "All relevant data are within the manuscript and its Supporting Information files." However, no original data or description regarding original data access is mentioned in the manuscript, except for the summary and model statistics based on the original data. PLOS ONE has a strict policy regarding data availability. It's crucial for the authors to address this major issue accordingly.

2.

It's not rare to observe that low eGFR tends to correlate with high urinary albumin-creatinine ratio (UACR) in clinical studies regarding patients with CKD. Even though this study focuses on patients with no diagnosed CKD using the provided definition, it's still recommended to add a little discussion to clear potential concerns over multicollinearity between eGFR and UACR, as they are both used in the linear regression model. Potential supporting evidences include the Pearson/Spearman Correlation Coefficient or the Variance Inflation Factor (VIF).

3. "The regression model did not include Cr because of the collinearity between Cr and eGFR."

In the Statistical analysis section of Materials and Methods, it's claimed that there exists a strong correlation between Cr and eGFR. However, it's very strange that Cr is significantly different between the male and female groups in Table 1, while eGFR shows almost no difference between those two groups. Is the "eGFR" actually the "Urinary albumin–creatinine ratio"? Please double-check.

4.

It's mentioned in the Statistical analysis section of Materials and Methods that the Student's t-test was used to compare the continuous variables between the male and female groups. In this case, because means and variances are directly used in the Student's t-test and p-values are also derived from it, it's highly recommended to use means and 95% confidence intervals (CI) instead of medians and quartile ranges in Table 1. This also enhances the understanding of why some variables are significantly or marginally significantly different between the male and female groups, as the current statistics do not always seem to facilitate each other.

5. "20.2-74.8"

In Table 1, the third quartile of BMI for the female group is shown to be "74.8". This number is very suspicious, given that the corresponding number from the male group is only "25.9" and there is no significantly higher percentage of diabetes mellitus observed in the female group. Please double-check this number.

Wording

1. **“Furthermore, adapting IS in clinical practice is problematic because it’s primarily measured through high-performance liquid chromatography.”**

In the Abstract section, it’s a little unclear what “adapting” means in this sentence. If it means measuring, but “adapting” is used instead of “measuring” to avoid repeating because “measured” is used later, it’s recommended to use words like quantifying or evaluating. If it means adding IS evaluation to regular CKD clinical practice, it’s recommended to use words like incorporating or adopting. Any other words that make the statement clearer are also perfectly acceptable.

2. **“Key Words: indoxyl sulfate, normal renal function, renal function markers”**

It’s more common to use “Keywords” instead of “Key Words”.

3. **“In 2017, the estimated prevalence of chronic kidney disease (CKD) was 9.1% worldwide, which was approximately 30% higher than in 1990. The estimated number of deaths is 120 million, with cardiovascular disease-related deaths exceeding 140 million [1].”**

In the Introduction section, the verb tenses are not consistent. In the first sentence, “was” is used, while “is” is used in the sentence after that. It’s highly recommended to re-examine the wording and keep the tenses consistent.

4. **“When disease-specific conditions damage the kidney, decreased glomerular filtration due to the reduced number of functioning nephrons.”**

In the sentence from the Introduction section, a verb is missing after “decreased glomerular filtration”, such as “occurs”.

5. **“In the present study, constipation was defined as less than once every 2 days and the participants were categorized into the non-constipation group, defined as those with defecation more than once a day, or the constipation group, defined as those with defecation less than once every 2 days.”**

In the Study setting and participants section of Materials and Methods, two categories are mentioned regarding the constipation group categorization. However, it’s unclear how a category is assigned for subjects with average defecation frequencies of less than once a day but more than once every two days. Please consider making the definition clearer.

6.

In Table 1, it’s recommended to change the header “***P***” to “*p-value*” to be consistent with the format of Table 2.

7.

In Table 1, the percentage format of the prevalence of hypertension for the male group should be consistent with other formats in the same table. Please keep one decimal place.

8.

In the footnote of Table 1, the first three lines might have a different font size from the last line. It's recommended to double-check.

9.

In addition, it's better to keep Table 1 and 2 formats consistent. For example, all cells in Table 1 are presented in bold fonts regardless of whether the p-values are significant or not, while only the significant p-values themselves in Table 2 are in bold fonts.

10. "The median IS level in the participants was 3.7 (IQR: 2.7–5.0) µmol/L, with males having an IS level of 3.85 (IQR: 2.6–5.4) µmol/L and females having an IS level of 3.7 (IQR: 2.8–4.8) µmol/L (Fig 2)."

In the Serum IS concentration section of Results, "3.85" has one more decimal place than all other numbers reported in the same sentence. Please consider making it consistent with others, including changing it accordingly in Figure 2 and its legend. This issue is also observed elsewhere. Please revise all the number formats where applicable.
