# Supplementary material for: The evaluation of indoxyl sulfate in the general population in Kanegasaki Iwate: A cross-sectional study (KANEGASAKI study)
Source: PLoS One. 2025 Dec 17;20(12):e0332655. doi: 10.1371/journal.pone.0332655 (PMC12711065; doi:10.1371/journal.pone.0332655)
Supplement: Attachment — Submitted filename: PONE-D-25-26168_R1 Comments.pdf [file pone.0332655.s009.pdf]

PONE-D-25-26168_R1 Comments

Thanks for the authors' responses. Undoubtedly, the authors have reviewed all reviewer comments carefully and resolved most of my concerns. I just have a few follow-up comments based on my previous ones that would need the authors' attention.

1. **Data S1. Anonymized data files required to reproduce the analysis - original data. (CSV)**

Somehow, I could only access the "Suppl Table.pptx" file in the downloading link of Supporting Information. However, it was claimed that a CSV file with the original data was also uploaded, as written in both the manuscript and the authors' response. It could be that the downloading link from my side might not have been updated. But it will be crucial for the authors to confirm that the original data can actually be downloaded using the downloading link in the manuscript.

2. **KeyWords: indoxyl sulfate, normal renal function, renal function markers**

Just like "Research" should never be written as "ReSearch", it is inappropriate to use "KeyWords" instead of "Keywords". Please revise it.

3. **In the present study, constipation was defined as at most once every 2 days and the participants were categorized into the non-constipation group, defined as those with defecation more than once a day (≥1 time /day), or the constipation group, defined as those with defecation at most once every 2 days (≤1 time / 2days).**

I think the authors were confused about my previous comment on the constipation definition. I was thinking that the current definition was unclear in some scenarios. For example, if a subject had 4 defecations on different days of the past week, that would result in a defecation frequency of 4 / 7 = 0.57 per day. This value is neither ≥1 time/day nor ≤1 time/2days, and thus it's confusing how this scenario should be classified.

However, the data collection method might be specifically designed such that no confusion would be possible. For example, if subjects were asked how frequent their defecation was, with provided options like "daily", "once every two days", and "once more than two days", the current definition would make sense. It will be helpful if the authors could bear in mind that there will be readers thinking like me in the way shown in the first paragraph, and try to avoid confusion in this case.

4. **Figure 2**

Somehow, there is a grey text box with Japanese characters in Figure 2. Please double-check if it was accidentally placed there or intentional.
