# Supplementary material for: The evaluation of indoxyl sulfate in the general population in Kanegasaki Iwate: A cross-sectional study (KANEGASAKI study)
Source: PLoS One. 2025 Dec 17;20(12):e0332655. doi: 10.1371/journal.pone.0332655 (PMC12711065; doi:10.1371/journal.pone.0332655)
Supplement: Attachment — Submitted filename: Response to Reviewer.docx [file pone.0332655.s008.docx]

**Response to Reviewer 3**

1. Were these factors assessed in the study? hydration status, physical activity, and time of daythey are known to affect the ACR level in addition to other factors.

**Response:** We thank the Reviewer for their salient inquiry. According to the GBD Chronic Kidney Disease Collaboration, body size, posture, pregnancy, exercise, fever, and activation of the renin-angiotensin system are associated with albumin excretion. Generally, renal function is determined using the protein/creatinine or the albumin/creatinine ratio calculated in spot urine samples because of difficulties encountered during urine collection (Abstract, page 2, line 23).

2. Why diet and gut microbiota were not accounted to in this study? They are known to affect IS levels.

**Response:** The Reviewer raises a valid concern. Indeed, indole, a precursor of IS, is produced by the gut microbiota and the serum levels of IS may be influenced by diet and intestinal flora (references 3 and 4) (Abstract, page 2, line 29). Although we did not evaluate the impact of diet or intestinal flora on serum IS levels, we wholeheartedly agree with the Reviewer and will consider this aspect in future studies

3. add and

**Response:** We thank the Reviewer and apologize for the oversight. The word “and” has been added (Abstract, page 2, line 39).

4. Is this the interquartiule range (IQR)?:

**Response:** We thank the Reviewer for their suggestion. Indeed, we reported the interquartile range. The revised Abstract has been revised accordingly (Abstract, p.2, lines 42).

5. If the enzymatic IS measurement method quantifies the total IS, the 3.7 umol/L is still within the normal range of serum IS levels.how many of the participants showed IS higher than 6.3 umol/L?

**Response:** We thank the Reviewer for their important question. The serum IS levels were above 6.3 µmol/L in 92 participants in our study.

6. High light
7. Add keywords.

**Response:** We thank the Reviewer for their careful observation. Relevant keywords have been added (Abstract, page 3, lines 50–51).

8. I suggest to summarize these statments and use at the negining of your abstract to help the readers get inot the scientific field addressed in this manuscript.

**Response:** We thank the Reviewer for their suggestion. We believe that these sentences only show the prevalence of CKD and future projections and do not necessarily represent the content of our study. Therefore, statements regarding CKD prevalence were excluded from the Abstract, which also allowed us to conform to the word count limitations (Abstract page 3, lines 42–45; Introduction page 4, lines 53–57).

9. Across the manuscrip, please change the writing style from the first point 0o fview (we, you, etc) to the third point of view (it is anticipated that etc...)

**Response:** We thank the Reviewer for their suggestions. As recommended, we have changed the writing style from the first point of view to the third point of view (Introduction page 4, line 57).

10. Same

**Response:** We thank the Reviewer for their suggestions. We have changed the writing style from the first point of view to the third point of view in this instance as well (Introduction page 4, line 70).

11. Citation? Are all these statements derived from citation 8 Niwa T, Ise M. Indoxyl sulfate, a circulating uremic toxin, stimulates the progression

**Response:** We thank the Reviewer for their inquiry. Indeed, all these statements were derived from the study cited in reference #10 (Introduction pages 4–5, lines 71–78).

12. L
13. remove "

**Response:** We thank the Reviewer for their suggestions. Accordingly, we have removed “in our manuscript. (Introduction page 6, line 102).

14. L
15. This statement is not clear. requires revision and clarification.

**Response:** We thank the Reviewer for their salient feedback. We have revised the statement for improved clarity (Materials and Methods page 7, lines 124–126).

16. Please revise this classificaiton of defecation frequency. Honkura et al. 2016 explicitly descrive the CVD HR in participants concerning their defecation frequency. They used more than or eaqual to 1 time per day as reference, whcih indicates that this is the normal classification in their consideration. it is very important to restratify your data on defecation frequency according to the citation you have listed. a recommendation is to calculate the number of defecations per week and to do the classification accordingly.normal, more than or equal to 7 time per week not normal less than 7 times per weekanother suggestion is to use the number of defecation as a continuous variable in your regression model

**Response:** We thank the Reviewer for their recommendation. We agree with the Reviewer’s suggestion. Although we were not able to classify the participants according to the study recommended by the Reviewer, we used a questionnaire to determine the frequency of defecation in our study (Materials and Methods page 7, lines 129–134).

17. Unify the abbreviation of creatinine

**Response:** We thank the Reviewer for their careful observation. Accordingly, the abbreviation for creatinine was revised from Cre to Cr (Material and Methods page 8, line 145).

18. redundant. please remove since the details is mentioned later.

**Response:** We thank the Reviewer for their suggestion. The sentence has been removed (Results page 8, line 157).

19. Were there any participants with total Serum IS levels higher than 6.3 umol/l? Normal range derived form DOI: 10.1007/s12012-014-9270-2

**Response:** We thank the Reviewer for their important question. The serum IS levels were above 6.3 µmol/L in 92 participants in our study. This study was cross-sectional study, and the result of the linear regression analysis alone could not define the normal range. Therefore, we did not present the normal range in the present study.

20. Why the authors choose these variables for their model. they did not mention that these variables are statistically significant at the bivariate level with IS levels.otherwise, the authors should present the literature evidence of the scientific significance of their choice of variables in the modle. The authors did not report on the use of the antibiotics in the participants. IS is derived from microbiota and antibiotics should be considered for any study related to IS.

**Response:** We thank the Reviewer for their suggestion. The outcome involved continuous variables, and linear regression was used to evaluate the association of serum IS with the variables of interest. The present study was based on data collected during a health checkup, and data on antibiotics were lacking. We acknowledge this aspect as a study limitation (Discussion, page 13, line 263).

21. It seems there might be a misunderstanding or a typo because the coefficient estimate (0.087) should be within the confidence interval range. If the confidence interval is from 0.002 to 0.011, the coefficient estimate should logically fall within this range.please review all the estimates reported in the tables 2 and 3 and their corresponding CIs. Please consult a biostatistician to run the linear regression and to validate the reported values in the tables.consider running a conditional stepwise regresion model

**Response:** We thank the Reviewer for their helpful suggestions. In Table 2, the numbers were marked with standardized partial regression coefficient estimates whereas the regression coefficient estimate was not marked. And the items 95% confidence interval of eGFR was typographical error. The data presented in Tables 2 and 3 were changed from standardized partial regression coefficients to 95% confidence intervals to unify the statistical concept. Fumiaki Takahashi, one of the authors, is a statistician and has confirmed the statistical methods and results following the recommendation of the Reviewer (Table 2, page 11, line 216).

22. Beta outside the CI

23. Beta outside the CI

**Response:** We thank the Reviewer for their careful observation. The standardized partial regression coefficients presented in Table 2 have been changed to 95% confidence intervals to unify the statistical concept (Table 2, page 11, line 216).

24. Frequency of defecation was used as a catigorical variable according to the definitin used in the method section, right. remove the catigorization and run the model on the continuous defecation frequency variable.it is highly recommended that the authors run a conditional regression model (forward stepwise) so they can have a better estimation of the association

**Response:** We agree with the Review. However, a questionnaire that employed distinct categories were used to evaluate the frequency of defecation in the present study. Therefore, we were unable to analyze the continuous defecation frequency that was recommended by the Reviewer (Materials and Methods page 7, lines 129–134).

25. Is this confidence interval right? if the association is significant, it should not be passing through zero.please review all your CI and validate them. consult with a biostatistician to review the tables and CIs.

**Response:** We thank the Reviewer and apologize for the oversight. The original presentation “0.007, 0.013,” was a typographical error and was missing a minus sign. The correct 95% CI is “−0.007, −0.001”. This has been corrected in the revised Table (Table 2, page 11, line 216).

26. Beta is outside the CI

27. Beta is outside the CI

28. The estimate should be within the CI. review and consult a Biostat

29. How come this interval is passing through zero and the p value is significant

**Response:** We thank the Reviewer for their suggestion as well as their comment on Table 3 (26-29). Fumiaki Takahashi, one of the authors, is a statistician who has confirmed the statistical methods and results following the recommendation of the Reviewer. We have removed the linear regression analysis with eGFR as the objective variable, because the inclusion of this analysis rendered it difficult to understand the study goal. We have also corrected the result “-5.851, 1.142,” which was a typographical error with a missing minus sign, to “−5.851, −1.142”. Moreover, the data presented in Table 3 have been changed from standardized partial regression coefficients to 95% confidence intervals to unify the statistical concept.
